# Protocol for the development of the WHO package of ear and hearing care interventions

**DOI:** 10.1136/bmjph-2025-002852

**Published:** 2025-05-19

**Authors:** Lauren K Dillard, Pallavi Mishra, Carolina Der, Shelly Chadha

**Affiliations:** 1 Department of Noncommunicable Diseases, World Health Organization, Geneva, Switzerland; 2 Otolaryngology - Head and Neck Surgery, Medical University of South Carolina, Charleston, South Carolina, USA

**Keywords:** Public Health, Epidemiology, Preventive Medicine

## Abstract

**ABSTRACT:**

**Introduction:**

This protocol article describes the methodology that guides the development of the package of ear and hearing care interventions (PEHCI), led by the WHO. The PEHCI aims to provide countries and other end-users with information related to evidence-based ear and hearing care (EHC), which can be used to prioritise interventions and plan for the resource requirements and costs to facilitate the implementation of the interventions into national health services packages and policies. The PEHCI can serve as an important resource towards strengthening EHC within health systems, ultimately allowing more people to benefit from EHC interventions.

**Methods and analysis:**

The development of the PEHCI comprises four phases. The first is to select priority EHC conditions through literature reviews and consultation with experts. The second is to identify evidence-based EHC interventions corresponding to those conditions, by reviewing high-quality clinical practice guidelines and systematic reviews. The third is expert agreement on interventions and their service delivery platforms, and to create descriptions for resource requirements for each intervention. The fourth is peer review. The project will be led by the WHO EHC Programme and will be supported by relevant stakeholders throughout the development process, including experts in EHC research and practice.

**WHAT IS ALREADY KNOWN ON THIS TOPIC**

- Hearing loss is an important global public health concern, yet interventions related to ear and hearing care are poorly integrated into healthcare in many low- and middle-income countries.

**WHAT THIS STUDY ADDS**

- The package of ear and hearing care interventions, led by the WHO, will provide evidence-based recommendations to support countries in their decision-making towards identifying priority ear and hearing care interventions, and planning for resource requirements and costs to facilitate the implementation of the interventions into national health services packages and policies.

**HOW THIS STUDY MIGHT AFFECT RESEARCH, PRACTICE OR POLICY**

- The package of ear and hearing care interventions will be a resource to aid in strengthening ear and hearing care within health systems, which ultimately could allow more people to benefit from critical ear and hearing care interventions.

## Introduction

 Hearing loss is a global public health priority given its high prevalence and meaningful impacts on individuals and society.^1 2^ Although interventions to address ear and hearing care (EHC) conditions are cost-effective and feasible,^2 3^ there are persistent global inequities in their access.^4^ These inequities are most pronounced in low- and middle-income countries (LMICs), where hearing loss is most common, and its impacts are most pronounced.^4^ Major barriers that inhibit access to EHC services include high service costs and a global dearth of qualified EHC providers.^4^ To overcome these barriers, health systems must be strengthened using a primary healthcare approach. That is, EHC services must be integrated across different levels and sites of care.^2^

Integration of EHC services is key to achieving universal health coverage, which ensures all people receive quality and accessible health services without suffering financial hardship.^5 6^ Key public health interventions for EHC provision are summarised in the acronym ‘H.E.A.R.I.N.G.’: (H): hearing screening and intervention; (E): ear disease prevention and management; (A): access to technologies; (R): rehabilitation services; (I): improved communication; (N): noise reduction; (G): greater community engagement. Over 10 years, implementing H.E.A.R.I.N.G. interventions would benefit nearly 1.5 billion people globally, avert 130 million disability-adjusted life years and bring productivity benefits exceeding US$2.4 trillion.^1^ The 10-year return on investment of implementing H.E.A.R.I.N.G. interventions would return US$16 for every $1 invested.^2^ Each country must determine which H.E.A.R.I.N.G. interventions require priority to be integrated into their national health services.

To support countries in determining the priority EHC interventions to include, WHO aims to provide guidance on the most relevant conditions to address across levels of care (primary, secondary, tertiary) and the resources required. To this end, WHO will develop a package of evidence-based EHC interventions to assist countries in making decisions regarding which interventions to prioritise, how to budget for these interventions and how they can be integrated into national health services packages and policies. The purpose of this article is to describe the methodology that guides the development of the package of EHC interventions (PEHCI), which can serve as an important resource towards strengthening EHC within health systems, ultimately allowing more people to benefit from EHC interventions.

## Methods and analysis

**Figure 1:**
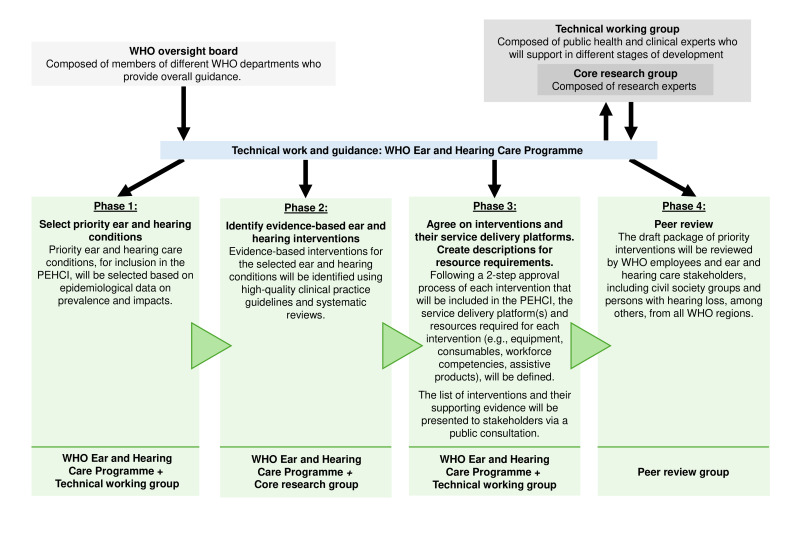
An overview of the stepwise approach that will be used to develop the package of ear and hearing care interventions (PEHCI).

Development of the PEHCI will follow the approach shown in figure 1 and will engage stakeholders throughout the process. Development will follow similar processes to those for the WHO package of interventions for rehabilitation and package of eye care interventions.^7^,^10^ The WHO EHC Programme will coordinate the project and lead the technical and development work. A WHO oversight board, composed of WHO members from different departments, including the WHO Guideline and Review Committee Secretariat, will oversee the project. This work is planned to occur between April 2024 and December 2025.

The technical working group will be composed of external (non-WHO) EHC experts in public health and clinical practice, from all six WHO regions and from countries at all income levels, who will provide guidance in the development of PEHCI. A subset of the technical working group, the core research group, composed of research experts in EHC, will provide research-based support. The roles of these groups are detailed later. Briefly, these expert groups will collaborate with WHO to identify evidence on EHC conditions requiring intervention across levels of care, and subsequently, will identify key interventions and create descriptions of resources required for the provision of each intervention. Finally, as discussed later in this protocol, a broader group of peer reviewers, consisting of stakeholders from all six WHO regions, including civil society groups and persons with hearing loss, among others, will be consulted for their feedback in a later stage of the development of the PEHCI.

The declaration of interests of all external contributors will be managed by the technical lead of the WHO EHC Programme, and where needed, assessed by an ethics officer from the Office of Compliance, Risk Management and Ethics at WHO. If a conflict of interest exists, the ethics officer will advise on which can be managed and which preclude participation.

### Phase 1. Select priority EHC conditions

The first phase of development involved the selection of EHC conditions for which interventions will be included in the PEHCI. The WHO EHC Programme performed the initial selection of conditions based on: (1) epidemiological data, including global and/or regional prevalence estimates, on causes of EHC conditions or impairments among children and adults; (2) epidemiological data on the impacts of EHC conditions or impairments among children and adults and (3) expert opinion on EHC conditions amenable to interventions in primary care settings.

Each technical working group member independently reviewed the list of conditions and provided written feedback on the list and the collated data. The initial draft of priority EHC conditions is presented in table 1. This list is not final and will be reviewed and finalised following literature reviews and discussion with experts.

**Table 1:** Initial draft of priority ear and hearing conditions to be considered for inclusion in the package of ear and hearing care interventions (PEHCI)

Ear or hearing condition	Brief description
**External ear**	
Foreign body in ear	Foreign body that is in the ear
Impacted cerumen	Hard plug of impacted wax/cerumen in the external ear
Otitis externa	Infection of external auditory canal
Structural developmental anomalies of ear causing hearing impairment	Abnormal developmental formations of the ear
**Middle ear**	
Acute otitis media	Suppurative and nonsuppurative ear conditions characterised by inflammation of the inner ear, accompanied by acute infection
Chronic otitis media	Suppurative and nonsuppurative ear conditions characterised by inflammation of the middle ear and that occur chronically
Cholesteatoma of middle ear	Abnormal growth of skin cells in middle ear or mastoid process
Otosclerosis	Abnormal bone growth inside the ear of unknown cause, with possible genetic and environmental influences
Traumatic rupture of ear drum	Damage (often perforation) of tympanic membrane caused by acute trauma
**Inner ear**	
Acquired hearing impairment	Loss of hearing that occurs sometime during the course of life and is not present at birth
Congenital hearing impairment	Hearing loss present from birth
Meniere disease	Disorder of inner ear causing vertigo, tinnitus, hearing loss and aural fullness
Noise effects on inner ear	Hearing loss caused by acute or chronic exposure to loud noises or recreational sounds
Ototoxic hearing loss	Hearing loss caused by exposure to ototoxicants, commonly certain types of medications
Presbycusis	Hearing loss associated with ageing
Sudden idiopathic hearing loss	Hearing loss that is characterised by a sudden onset
Tinnitus	Persistent sensation of ringing or buzzing in the ear

Terms are consistent with the International Classification of Diseases, 11th revision (ICD-11).

### Phase 2. Identify evidence-based EHC interventions

The WHO EHC Programme, supported by the core research group, will identify evidence-based EHC interventions to be included in the PEHCI through high-quality clinical practice guidelines and systematic reviews. Clinical practice guidelines will be the main source of evidence used to identify evidence-based EHC interventions. Clinical practice guidelines are highly valuable because they (1) cover the continuum of clinical care for a given EHC condition, (2) combine scientific evidence with clinical expertise and (3) cover evidence gaps of published literature through expert consensus procedures. As needed, high-quality systematic reviews will complement the evidence from clinical practice guidelines. Processes for selecting or conducting systematic reviews are discussed later. The conduct of all systematic reviews, including those to identify clinical practice guidelines and newly conducted systematic reviews, will be overseen by the WHO EHC Programme, including experts in systematic review methodology, and will be reported using Preferred Reporting Items for Systematic Reviews and Meta-Analyses (PRISMA) guidelines.^11^

#### Identification of clinical practice guidelines

The core research group will conduct a systematic literature search of guideline databases and selected academic databases for each condition identified in phase 1. Websites for professional otolaryngology and audiology associations will also be searched for relevant guidelines. All processes related to the systematic literature reviews, including the development of search strings, will be conducted under the guidance of the WHO EHC Programme. As necessary, search terms and filters will be adapted to effectively conduct the search across different databases and websites. Searches will identify clinical practice guidelines published from 2014 onwards, to ensure interventions reflect current evidence-based research and practice, and in several languages, including English, Spanish, French, Russian, or Chinese.

Two members of the core research group will serve as reviewers and will independently screen the titles and abstracts of the clinical practice guidelines. The following exclusion criteria will be applied: (1) the identified literature is not a guideline and (2) the guideline is not related to the selected EHC condition. The same two reviewers will perform a full-text screen of clinical practice guidelines. Guidelines will be excluded if there is a (1) presence of commercial funding and/or unmanaged conflicts of interest of contributors and/or (2) absence of affiliations of all contributors. Any disagreements related to the inclusion/exclusion of guidelines will be resolved by a discussion between the two reviewers and will involve a third reviewer as needed.

After the full-text screening, the same reviewers will independently evaluate the quality of the clinical practice guidelines using the Appraisal of Guidelines for Research and Evaluation (AGREE II) tool.^12^ Nine AGREE II items (numbers 4, 7, 8, 10, 12, 13, 15, 22 and 23) will be used to evaluate the quality of the guidelines.^7 9^ For a given guideline, if the rating of an item differs by more than two points between the two reviewers, they will discuss the results and involve a third reviewer, as needed, to reach consensus. Guidelines will be excluded if the reviewers rate the average score for items 4, 7, 8, 12 or 22 below 3 points, and if the sum of the average score for all nine items is less than 45 points.^7^

A maximum of five clinical practice guidelines will be selected for each EHC condition. If there are guidelines for specific age groups for a given condition (eg, children, youth, adults), up to five clinical practice guidelines will be selected for each age group. If more than five guidelines are identified for a given EHC condition, the selection of clinical practice guidelines will be determined via consensus procedures among members of the WHO EHC Programme (with or without input from technical working group members). Consensus will be achieved by considering relevant information, including AGREE II scores, date of publication and representativeness of the clinical practice guideline, in terms of country, regional or international scope of the guidelines. These processes ensure the selection of high-quality clinical practice guidelines while reducing possible redundancies in the content of the guidelines.

#### Identification or conduct of systematic reviews

Evidence from high-quality systematic reviews will be used to complement evidence from clinical practice guidelines in the following cases: (1) no clinical practice guidelines are identified for a given EHC condition after screening and quality appraisal, (2) the selected clinical practice guidelines provide contradictory recommendations for a given intervention and/or (3) the clinical practice guideline makes recommendations for an intervention based on evidence that is published prior to 2014. Systematic reviews will be identified by the core research group, under the guidance of the WHO EHC Programme. The identification or conduct of systematic reviews is meant to overcome limitations in the availability of clinical practice guidelines.

First, Cochrane systematic reviews, published or updated since 2014, will be identified. If Cochrane systematic reviews for a given intervention do not exist, other high-quality systematic reviews will be identified through a systematic search process. To ensure the selection of high-quality systematic reviews, two reviewers will independently appraise the quality of the systematic review methodology using the A MeaSurement Tool to Assess Systematic Reviews 2 (AMSTAR 2).^13^ All non-Cochrane systematic reviews will (1) be screened on AMSTAR 2 item 2 (whether the report of the review contained an explicit statement that the review methods were established prior to the conduct of the review) and (2) when item 2 was rated ‘yes’, reviewers will assess the remaining critical domains. Systematic reviews will be included if all critical item domains (items 2, 4, 7, 9, 11, 13, 15) are rated as ‘yes’ or ‘partial yes’ by both reviewers.^7 9 13^

Like the approach described earlier, two members of the core research group will independently perform the title and abstract, then the full-text screening, to determine inclusion of systematic reviews. Systematic reviews will be excluded if they do not focus on an intervention or diagnostic test accuracy specifically related to the target EHC condition. Any disagreements related to the inclusion/exclusion of review articles will be resolved by a discussion of the clinical practice guidelines and will involve a third reviewer as needed.

If there are inadequate evidence-based EHC interventions identified from high-quality clinical practice guidelines or systematic reviews for a given condition, the core research group, under the guidance of the WHO EHC Programme, will conduct systematic reviews for those conditions. These systematic reviews will follow pre-defined protocols and will be reported following PRISMA guidelines.^11^

#### Data extraction and preparation

One reviewer will complete data extraction, which will be checked by the second reviewer. For clinical practice guidelines, data extraction will include the following: (1) information on the guideline (title, authors, year of publication), (2) reference to the recommendations, interventions and related outcomes, (3) content and strength of the recommendations and (4) quality of evidence related to the recommendations. For systematic reviews, data extraction will include the following information: meta-study information (title, authors, year of publication), information on the population, intervention and control, setting, statistical values for each analysis, number of studies and sample characteristics, summary of main results, certainty of evidence and adverse events.

### Phase 3. Agree on interventions and their service delivery platforms: create descriptions for resource requirements

Composed of members from the technical working group, subgroups of members with expertise related to each EHC condition (selected in phase 1) will be formed. Each member will have working or research experience in the provision of EHC interventions. Their primary role is to agree on whether the identified interventions should be retained in the package, whether there are relevant interventions that were not included, the service delivery platforms and the workforce competencies, time and resources required to provide each of the selected interventions. This process will consist of an online preparatory questionnaire, data preparation by WHO and a web-based group discussion among members of each subgroup applying pre-determined inclusion and exclusion criteria. Inclusion criteria for interventions are: (1) interventions are supported by high-quality guideline/s (assessed by the AGREE II tool) or high-quality literature reviews (published work or literature developed by core research group) and (2) interventions are approved by the majority of the technical working group members. Exclusion criteria for interventions are: (1) interventions are not supported by evidence (high-quality evidence could not be identified or identified evidence did not support the intervention) and (2) interventions are not approved by the majority of the technical working group members.

#### Online preparatory questionnaire

Each member will participate in an online questionnaire specific to their subgroup. This questionnaire will be developed by the WHO EHC Programme, and the components of the questionnaire are described later. Before participating in the questionnaire, members will receive full texts of the clinical practice guidelines and/or systematic reviews and the data extracted from them. For each intervention, group members will confirm whether they recommend the intervention for inclusion in the PEHCI based on the strength and quality of the recommendation, and its applicability and appropriateness in the settings in which they work. For each intervention the group members recommend for inclusion in the PEHCI, they will then assign each intervention to one or more service delivery platforms (eg, school, outreach, hospital, clinic, home). Group members will have the opportunity to identify missing interventions that were not included in the PEHCI but are considered relevant to the EHC condition.

#### Data preparation

After all questionnaires are completed, the WHO EHC Programme will collate the list of interventions for which majority agreement was achieved during the online questionnaire, and a list for which majority agreement was not achieved (and requires further discussion). Systematic literature searches will be performed for any missing interventions identified by each subgroup. If evidence supports the intervention, it will be added to the list of interventions requiring discussion during the web-based group discussion (described later).

The WHO EHC Programme will draft descriptions related to relevant resources for each intervention that achieved a majority consensus during the online questionnaire. This will include information on the target population, workforce requirements, most appropriate service delivery platform(s) (based on questionnaire results), time required to provide each activity/procedure related to the intervention and required health products, including equipment, consumables and assistive products.

#### Web-based group discussions

Results from the online questionnaire and the additional data collated by WHO (data preparation stage) will be presented to members of each subgroup prior to a web-based group discussion. The objective of the web-based group discussion is twofold. The first is to confirm the list of interventions to be included in the PEHCI. This involves achieving consensus on the interventions that did not achieve majority agreement for inclusion in the PEHCI during the online questionnaire, and the interventions that were identified as missing during the online questionnaire and are supported by evidence (determined from the systematic literature searches described earlier). The second objective is to review and update (if needed) the data collated by the WHO EHC Programme related to the service delivery platforms, equipment and workforce requirements for interventions that achieved consensus during the online questionnaire. Discussions will be held to identify overlaps in the provision of resources and to identify cross-cutting interventions that are relevant to the diagnosis, management and treatment of multiple EHC conditions.

Before the final stage of peer review, the list of interventions and their key supporting evidence will be presented to stakeholders via a public consultation. Feedback will be presented to the technical working group and incorporated into the PEHCI draft.

### Phase 4. Peer review

A peer review group composed of WHO employees and EHC stakeholders, including civil society groups and persons with hearing loss, among others, from all WHO regions will be formed. Peer review group members will independently review the draft of the PEHCI, which will include a list of interventions with core data and will provide feedback and recommendations for revision. All feedback and recommendations will be considered before producing the first version of the PEHCI.

### Future directions

On finalisation of the PEHCI, it will be integrated into the WHO universal health coverage compendium of interventions and linked to the WHO OneHealth Tool.^14 15^ These tools aim to inform national strategic health planning in LMICs by providing a framework for planning, costing, impact analysis, budgeting and financing, alongside details related to the population EHC needs. These resources provide countries and policy makers with an evidence-based, yet simple, approach to planning health interventions. The WHO EHC Programme will continue to work with other WHO programmes, such as ageing, vision and primary care, to ensure relevant details from the PEHCI are aligned and integrated with care packages of those programmes.

## Data Availability

Data sharing not applicable as no datasets generated and/or analysed for this study.
